# Old Apple Cultivars as a Natural Source of Phenolics and Triterpenoids with Cytoprotective Activity on Caco-2 and HepG2 Cells

**DOI:** 10.3390/foods13071014

**Published:** 2024-03-26

**Authors:** Kamil Szymczak, Małgorzata Zakłos-Szyda, Katarzyna Mietlińska, Adriana Eliašová, Iga Jodłowska, Daniela Gruľová, Grzegorz Hodun, Radosław Bonikowski

**Affiliations:** 1 Institute of Natural Products and Cosmetics, Faculty of Biotechnology and Food Sciences, Lodz University of Technology, Stefanowskiego 2/22, 90-537 Łódź, Poland; katarzyna.mietlinska@p.lodz.pl (K.M.); radoslaw.bonikowski@p.lodz.pl (R.B.); 2 Institute of Molecular and Industrial Biotechnology, Faculty of Biotechnology and Food Sciences, Lodz University of Technology, Stefanowskiego 2/22, 90-537 Łódź, Poland; malgorzata.zaklos-szyda@p.lodz.pl (M.Z.-S.); iga.jodlowska@p.lodz.pl (I.J.); 3 Department of Ecology, Faculty of Humanities and Natural Sciences, University of Prešov, 17, Novembra 1, SK-081 16 Prešov, Slovakia; adriana.eliasova@unipo.sk (A.E.); daniela.grulova@unipo.sk (D.G.); 4 Department of Variety Studies, Nursery and Gene Resources, Research Institute of Horticulture, Konstytucji 3 Maja 1/3, 96-100 Skierniewice, Poland; grzegorz.hodun@inhort.pl

**Keywords:** apples, antioxidants, polyphenols, triterpenoids, cell lines

## Abstract

Apples are among the most consumed fruits worldwide. They serve as an excellent source of compounds that have a positive impact on human health. While new varieties of apples are being developed, numerous varieties have been forgotten. In this article, we present the results of research on 30 old apple cultivars, focusing on both qualitative and quantitative determination of antioxidant properties, and content of total phenolics, phenolic acids, triterpenoids and polyphenols. Our analyses show significant differences in the total content of each group of compounds between apple cultivars, as well as the phytochemical profile. The richest source of antioxidants was revealed to be ‘Reneta Blenheimska’ and ‘Książę Albrecht Pruski’ varieties, but the highest amount of phenolics had ‘James Grieve’ and ‘Kantówka Gdańska’ (KG). Among studied apples KG, ‘Krótkonóżka Królewska’ and ‘Grochówka’ (G) were the richest source of phenolic acids and polyphenols, whereas G, ‘James Grieve’ and ‘Krótkonóżka Królewska’ had the highest level of triterpenoids. Based on these findings, we selected two cultivars, G and KG, for further in vitro cell line-based studies. Based on biological activity analyses, we demonstrated not only antioxidant potential but also proapoptotic and cytoprotective properties within human-originated Caco-2 and HepG2 cell lines. In the era of a dynamically growing number of lifestyle diseases, it is particularly important to draw the attention of producers and consumers to the need to choose fruit varieties with the highest possible content of health-promoting compounds and, therefore, with the strongest health-promoting properties.

## 1. Introduction

Apples are by far the most important of all cultivated fruits, both in Poland and Europe. Despite over 10,000 apple cultivars having already been grown, only a few of them have economic importance [1]. The reason for this situation is the global view of producers regarding the characteristics of the perfect apple: good appearance, sweet taste, and resistance to harvesting and transport conditions. Unfortunately, years of selective breeding have strongly influenced the content of valuable bioactive compounds. However, recently, there has been an increase in consumer reluctance to buy products created through selection or genetic modification, leading to a return to old cultivars of vegetables and fruits, which are considered healthier.

Considering the importance of daily consumption of vegetables and fruit for human health, apples are by far the best source of key nutrients, since they are cheap and easily available all year round. ‘An apple a day keeps the doctor away’ is a proverb originating in 19th-century Wales, suggesting that eating apples daily is an excellent preventive agent against diseases. There is a lot of truth in this saying because the list of health-promoting properties of compounds present in apples is extensive. Regular consumption of apples rich in phenolic compounds, tocopherols and carotenoids reduces the incidence of cancer, cataracts, chronic obstructive pulmonary disease, cardiovascular disease, asthma, type 2 diabetes, and the risk of stroke [2,3,4].

Apples are an extremely important source of phenolic compounds in our diet. Phenolics are responsible for most of the antioxidant activity in apple fruits, far exceeding the value that can be explained by the content of ascorbic acid alone [5,6]. Importantly, most of the mentioned health-promoting compounds are found primarily in the fruit’s skin. The flesh contains much lower amounts of catechins, procyanidins, chlorogenic and caffeic acids but is rich in sugars and vitamin C [2,7].

Recent research shows that phenolic compounds contained in apples can support neurogenesis, i.e., the process of creating new nerve cells. This has a positive effect on learning, memorizing, and overall improvement of brain functions. Quercetin has been shown to support the survival and differentiation of hippocampal precursor cells and induce endogenous antioxidants and the AKT signalling pathway (affecting cell survival, growth, proliferation, migration, and angiogenesis). Another compound, 3,5-dihydroxybenzoic acid, also present in apples, increases the proliferation and neurogenesis of precursor cells [8]. Moreover, consumption of flavanol-rich foods like apples is linked to lower chances of developing frailty, a geriatric syndrome that leads to a greater risk of falls, fractures, disability, and even mortality. Quercetin, which is particularly high in apples, shows a strong positive effect and correlates with lower odds of frailty [9]. Quercetin and other flavonoids in apples also have strong antiviral properties, acting by attaching to the surface of the virus particle, and thus preventing it from entering human cells or by inhibiting a number of viral proteins, and thus interrupting the virus multiplication cycle [10,11].

An extremely interesting, and quite poorly evaluated, group of compounds in apples are triterpenoic acids and their derivatives [12]. Ursolic and oleanolic acids are most abundant, but corosolic, maslinic, and betulinic acids are also important [13,14,15]. The last three of these, although they constitute quantitatively less than one-fifth of the sum of all determined triterpenic acids, are especially distinguished by much higher biological activity than the others. In addition, their alcohol derivatives present in apples, such as α- and β-amyrin, erythrodiol, uvaol, or betulin, also have significant health-promoting properties. Triterpenoids present in apples have various pharmacological properties: antimicrobial, antiviral, antifungal, antioxidant, immunomodulatory, anticancer, hemolytic, anti-inflammatory, hepatoprotective, and analgesic [16,17].

The aim of this study was to screen 30 selected European old apple cultivars for their most important active compounds and choose two with the highest content to test their cytoprotective activity on in vitro cell line models—human-originated enterocytes Caco-2 and hepatocytes HepG2.

## 2. Materials and Methods

### 2.1. Chemical and Reagents

All reagents were purchased from Sigma-Aldrich/Merck (Steinheim, Germany), unless stated otherwise. All cell culture reagents were purchased from Life Technologies (Carlsbad, CA, USA). The plastics used for the cell cultures were purchased from Greiner Bio-One GmbH (Frickenhausen, Austria). All measurements of biological activity were performed using the Synergy 2 BioTek Microplate Reader (BioTek, Winooski, VT, USA).

### 2.2. Samples

Apple fruits from 30 cultivars were collected in 2019 from the experimental field of Research Institute of Horticulture (Skierniewice, Poland) for this study (Table S1 in Supplementary Materials). Samples were carefully washed and dried with a paper towel. For each cultivar, three samples (i.e., mature apples picked from different parts of the same tree) were taken. For all analysis, apples were peeled, and peels were subsequently dried in 60 °C with air circulation for less than 24 h to maintain a constant weight. Finally, dried apple peel (DAP) was stored at −20 °C until extraction process.

### 2.3. Extraction Process

Dried apple peel (DAP) was ground with liquid nitrogen into fine powder. After warming to room temperature, 0.5 g of each sample was put into extraction thimbles, placed in 50 mL Soxhlet extractors, and poured with 60 mL of methanol. The process took about 40 min (6 cycles). Then, solvent was removed with rotary evaporator. The opened flasks were left overnight to release residual methanol. Finally, dry residues were weighed and reconstituted with 5 mL of methanol. Extracts were stored in glass screw cap vials at −20 °C until their analysis.

For the analysis of biological activity, apple peel extracts were diluted in methanol to obtain the concentration of 600 mg dry weight of the preparation/mL. For the experiments, the concentrated extracts were further diluted with culture medium so that a volume of 2 µL of the diluted preparation per 100 µL of culture medium was added to each test well. The range of tested extract concentrations was 0.1–3.75 mg/mL.

### 2.4. Antioxidant Properties

Antioxidant properties (AP) were determined using the 2,2-diphenyl-1-picrylhydrazyl (DPPH) method. It is a widely used spectrophotometric method with the DPPH reagent, which changes colour from dark violet to light yellow as a result of the reaction with compounds that possess antioxidant activity [18]. The colour change is observed at the wavelength between 515–517 nm. For the determination, a standard curve was prepared using solution of Trolox in methanol. 

To measure the total antioxidant capacity of the DAP extracts, 20 µL of the tested extract and 3 mL of DPPH was placed in test tubes and mixed vigorously, secured against oxygen, and placed in a dark place at room temperature for 30 min. After this time, the absorbance was measured at a wavelength of 517 nm. For each sample, three repetitions were made, and obtained values were converted to an equivalent amount of Trolox (mgTE/100 g of DAP).

### 2.5. Total Phenolic Content

Total phenolic content (TPC) analysis was performed using a relatively popular spectrophotometric method with the Folin–Ciocalteu (FC) reagent. It is based on the colour change of the FC reagent from colourless to blue-navy blue, with the maximum absorbance at the wavelength of approximately 750 nm, which has been well-described in the literature [19]. For the results, a standard curve was prepared using gallic acid in methanol 80%. To measure the TPC 0.125 mL of the tested DAP extract, 0.25 mL of FC reagent, 2.5 mL of 20% Na_2_CO_3_ was added to a 25 mL volumetric flask and filled up to the mark with distilled water. After vigorously mixing, samples were put it in a dark place for 30 min. After this time, the absorbance was measured at a wavelength of 750 nm. For each sample three repetitions were made, and the obtained absorbance values were converted to an equivalent amount of gallic acid (mgGAE/100 g of DAP).

### 2.6. GC–MS Analysis of Phenolic Acids

For the phenolic acids analyses, methanolic extracts were saponified and directly hydrolysed. The previously described method [20] was used, with changes only in the volumes of used reagents. Firstly, 0.5 mL of methanolic DAP extract was heated with 5 mL of methanolic NaOH solution (1 M). In the second step, 2 M aqueous HCl solution was added to obtain its concentration in the flask of about 0.5 M. After hydrolysis, methanol was evaporated from the sample, before 3 mL of ethyl acetate was added and shaken. For analysis, 0.2 mL of the ethyl acetate layer was moved into a 1.5 mL glass chromatographic vial and evaporated to dryness under a gentle stream of nitrogen. After evaporation, 0.2 mL of bis(trimethylsilyl)trifluoroacetamide with trimethylchlorosilane (BSTFA + TMCS) (99:1) was added and heated on a hotplate for 2 h at 80 °C. Gas chromatography coupled with mass spectrometry (GC–MS) analyses were performed on a LECO Pegasus 4D apparatus, equipped with an Agilent 7890A gas chromatograph, coupled with a mass spectrometer with a time-of-flight analyser with an adapted method previously described for phytosterols measurement [21].

### 2.7. GC-–MS Analysis of Triterpenoids

Triterpenoids derivatization protocol was developed based on methods described in the literature [13,22] with slight changes. More specifically, 100 µL of methanolic DAP extracts was transferred to 1.5 mL glass chromatographic vials and evaporated to dryness under a gentle stream of nitrogen. Then, 100 µL of pyridine and 100 µL of *N*,*O*- BSTFA + TMCS were added. The vials were screwed on tightly and placed on a heating plate with a temperature of 80 °C for 2 h. After this time, samples were left in a dark place at room temperature for 24 h. Before the analysis, the samples were transferred into micro inserts and placed on the tray of the Gerstel MultiPurpose Sampler autosampler. GC–MS analyses were performed on a LECO Pegasus 4D apparatus, equipped with an Agilent 7890A gas chromatograph coupled with a mass spectrometer with a time-of-flight analyser. The compounds were separated on a BPX5 column (30 m × 0.25 mm × 0.25 µm) from Trajan Scientific Australia Pty Ltd. The carrier gas was helium at a flow of 1.5 mL/min. The temperature program started at 250 °C for 1 min, followed by an increase of 15 °C/min to 320 °C and maintained for 15 min (total analysis time was 21 min). The inlet temperature was 310 °C. The mass spectrometer worked in the Electron Impact mode at −70 eV, and in the scanning range (*m*/*z*) from 33 to 750 amu. The temperature of the ion source was 200 °C, and the temperature of the transfer line was 280 °C.

### 2.8. HPLC Analysis of Polyphenols

Methanolic DAP extracts were filtered using syringe filters (17 mm, Nylon (PA), pore diameter 0.2 µm), transferred in HPLC screw cap vials and analysed. HPLC analyses were performed on a Dionex UltiMate 3000 Quaternary Analytical System with a DAD detector (Dionex Softron GmbH, Germering, Germany), on a Dionex Acclaim 120 C18 column (5 µm, 250 mm × 4.6 mm), at a temperature of 25 °C. The mobile phase consisted of solvent A (0.1% formic acid in HPLC gradient grade water, *v*/*v*) and solvent B (0.1% formic acid in HPLC gradient grade acetonitrile, *v*/*v*). The flow rate was 0.9 mL/min. The gradient elution program was as follows: 0 min, 5% B; 0–10 min, 5 → 20% B; 10–25 min, 20 → 23% B; 25–35 min, 23 → 50% B; 35–37 min, 50 → 100% B; 37–43 min, 100% B; 43–45 min, 100 → 5% B; 45–50 min, 5% B. Compounds were detected at wavelength 280, 320 and 350 nm. Identification of the compounds was performed based on the retention time and the absorption spectra recorded during the analysis by comparison to the following standards: chlorogenic acid, epicatechin, rutin (quercetin-3-*O*-rutinoside), hyperoside (quercetin-3-*O*-galactoside), isoquertitin isoquercitrin (quercetin-3-*O*-glucoside), quercitrin (quercetin-3-*O*-rhamnoside), and quercetin. Calibration curves for quantitative determinations were made for chlorogenic acid, epicatechin, quercetin and phloretin. Chlorogenic acid, although it belongs to the phenolic acids group, was analyzed with polyphenols.

### 2.9. Cell Culture

HepG2 cell line (human hepatocellular carcinoma) was obtained from the DSMZ (Deutsche Sammlung von Mikroorganismen und Zellkulturen) collection and cultured in a DMEM (Dulbecco’s Modified Eagle Medium) medium with 10% of fetal bovine serum (FBS), streptomycin (100 µg/mL), penicillin (100 IU/mL), fungizone (100 µg/mL). The Caco-2 cell line (human colon adenocarcinoma) was obtained from the ATCC (American Type Culture Collection) collection and cultured in DMEM medium with 10% FBS, streptomycin (100 µg/mL), penicillin (100 IU/mL), fungizone (100 µg/mL). The culture was conducted in an incubator at a constant temperature of 37 °C and humidity, in an atmosphere enriched with 5% CO_2_ concentration.

### 2.10. Metabolic Activity of Cells

The parameter was determined using the test with MTT (3-(4,5-dimethylthiazol-2-yl)-2,5-diphenyltetrazolium bromide) dye, which determines the activity of mitochondrial dehydrogenases that convert a soluble yellow MTT salt solution to a purple insoluble formazan. After the cells were incubated with the compounds, a standardized MTT solution (5 mg/mL) was added to the cells for 4 h; then the medium was removed and 75 µL of DMSO (dimethyl sulfoxide) dissolving formazan crystals was added. The absorbance was measured at 490 nm.

### 2.11. Detection of Intracellular Oxygen Free Radicals

The measurement was made using the probe DCF-DA (2′,7′-dichlorodihydrofluorescein diacetate), which is converted into a fluorescent product in the presence of radicals. After cell incubation with compounds, the medium was removed, and 100 µL of PBS (phosphate-buffered saline) buffer was added, followed by 10 µL of probe solution (10 mM DCF-DA). After 30 min incubation, the fluorescence F_ex_/F_em_ = 485/528 nm was measured.

### 2.12. ATP Level Measurement

After cell incubation with the preparations, 75 µL of the CellTiter-Glo^®^ assay (Promega GmbH, Walldorf, Germany) reagent was added to the medium. After 10 min, the luminescence was measured.

### 2.13. Externalization of Phosphatidylserine (PS)

After incubation with compounds, the cells were washed twice with PBS solution, then incubated with Annexin-V-FITC reagent (Annexin-V-FITC assay, 0.25 μg/mL) for 10 min. The fluorescence level was measured at the wavelength F_ex_/F_em_ = 485/530 nm. Cell membrane perforation was determined using propidium iodide (1 µg/mL), and after 30 min fluorescence was measured (F_ex_/F_em_ = 535/620 nm).

### 2.14. The Level of Lipid Accumulation

The level of lipid accumulation was determined using the Nile red probe, which acts as a hydrophobic fluorescent probe, peripherally dissolving and fluorescently labelling neutral lipid deposits inside the cell. After incubation, the cells were washed with PBS and fixed with 5% paraformaldehyde for 30 min. After rinsing with PBS, 1 µg/mL Nile red was added to a fresh 100 µL aliquot of PBS. After 40 min fluorescence was measured (F_ex_/F_em_ = 485/528 nm).

### 2.15. Glutathione Peroxidase (GPx) and Superoxide Dismutase (SOD) Activity

After cell incubation with preparations, the GPx (glutathione peroxidase) activity was measured using the Glutathione Peroxidase Assay kit (Cayman Chemical Co., Ann Arbor, MI, USA), whereas the SOD (superoxide dismutase) activity was measured using the Superoxide Dismutase Assay kit (Cayman) according to the manufacturer suggestions.

### 2.16. Statistical Analysis

Experimental data of the samples’ composition and relationships between them were examined using linear models (ANOVA, regression). These analyses were performed using Statistica 13.1 (StatSoft Polska, Kraków, Polska). Data are expressed as mean ± standard deviation (SD) for sample composition analyses or as mean ± standard error of mean (SEM) for biological activity results. A significance level of *p* < 0.05 was chosen (unless otherwise stated). Correlations of 0.5 or above are considered strongly significant by the authors. To maintain clarity of results full data, including SD/SEM, are presented in Supplementary Materials.

## 3. Results and Discussion

### 3.1. Content of Health-Promoting Compounds in Old Apple Cultivars

As a high antioxidant, properties (AP) value can be considered an equivalent amount of Trolox at the level of 1000 mgTE/100 g of DAP. This value was exceeded by 13 cultivars, i.e., in almost half of those examined (Table S2 in Supplementary Materials). Cultivars with the highest AP value were ‘Reneta Blenheimska’, ‘Książę Albrecht Pruski’ and ‘Jakub Lebel’ (1152.9, 1136.5 and 1129.8 mgTE/100 g of DAP respectively). In turn, relatively low AP values were found in ‘Kosztela’, ‘Grafsztynek Inflancki’ and ‘Kronselska’ (411.1, 481.0 and 536.9 mgTE/100 g of DAP respectively). The average value for all tested cultivars was 892.4 mgTE/100 g of DAP.

The highest TPC values were found in the cultivar ‘James Grieve’, ‘Grochówka’ and ‘Kantówka Gdańska’ (246.6, 217.0 and 214.2 mg GAE/100 g of DAP respectively). In turn, relatively low TPC values were found in ‘Malinowa Oberlandzka’, ‘Grafsztynek Inflancki’ and ‘Galloway Pippin’ (137.4, 143.2 and 144.7 mg GAE/100 g of DAP respectively). The average value for all tested cultivars was 173.2 mg GAE/100 g of DAP (Table S2 in Supplementary Materials).

Based on GC–MS analyses, eight phenolic acids were found in DAP extracts. seven compounds were identified and only one was classified to phenolics, due to the specific MS spectrum; however, its structure could not be accurately determined (Table S3 in Supplementary Materials). *p*-Coumaric acid and gallic acid were present in all apples at levels starting from detectable level (but below limits of quantification) up to 377.96 mg/100 g of DAP (Table S4 in Supplementary Materials). However, the highest individual content was calculated for ferulic acid in ‘Kantówka Gdańska’ and reached the content of 402.64 mg/100 g of DAP. Other studies also show extended variability in phenolic acids content between different cultivars [23]. The highest total value of phenolic acids was also found in the cultivar ‘Kantówka Gdańska’, with the content of 1661.03 mg/100 g of DAP. Other cultivars of note were ‘Krótkonóżka Królewska’, ‘Grochówka’ and ‘Boskoop’ (1153.67, 872.05 and 870.13 mg/100 g of DAP respectively). Several times lower content of phenolic acids were found in ‘Kosztela’, ‘Książe Albert’ and ‘Malinowa Oberlandzka’ (209.60, 210.41 and 221.86 mg/100 g of DAP, respectively). The average value for all tested cultivars was 620.97 mg/100 g of DAP.

Chromatographic analyses showed the presence of nine different triterpenoids, of which seven could be accurately determined using MS spectra and standards (Table S5 in Supplementary Materials). The triterpenoids that have been identified in all apple cultivars are oleanolic, ursolic and corosolic acids. Betulinic and maslinic acids were found in almost all cultivars (Table S6 in Supplementary Materials). Ursolic acid was characterized by the highest content among all triterpenoid acids (average 789.2 mg/100 g of DAP), followed by oleanolic acid (average 206.0 mg/100 g of DAP) and corosolic acid (average 149.2 mg/100 g of DAP). This results correlates well with other studies of triterpenoids in apples [14,24,25]. Among the apple cultivars, the highest total content of triterpenoids was found in ‘Grochówka’ (1976.8 mg/100 g of DAP), ‘James Grieve’ (1759.7 mg/100 g of DAP) and ‘Krótkonóżka Królewska’ (1751.5 mg/100 g of DAP). In turn, the lowest content was found in cultivars ‘Reneta z Brownlee’ (408.5 mg/100 g of DAP), ‘Reneta Kulona’ (648.3 mg/100 g of DAP) and ‘Galoway Pippin’ (732.0 mg/100 g of DAP). The average content of the sum of triterpenoids for all cultivars was 1234.2 mg/100 g of DAP. One variety, ‘Reneta z Brownlee’ is distinguished not only by the lowest content of the sum of triterpenoids, but also by its unusual composition. It has the lowest content of ursolic, oleanolic and corosolic acid from all the tested cultivars; and, at the same time, has the highest content of α-amyrin and betulinic acid, as well as distinctive content of betulin. As shown in studies, triterpenoid acids in apples are characterized by high differences in content [26]. For ursolic acid, an almost ten-fold difference was observed. In our studies, the spread of values is even greater, starting from 117.6, up to 1401.1 mg/100 g of DAP. Similarly, for oleanolic acid, an eight-fold difference was observed in the research and in our study, we found values from 43.4 up to 361.0 mg/100 g of DAP, also close to eight-fold differences.

For polyphenols analysis using the HPLC-DAD technique, all cultivars with total polyphenol content values above 200 mg GAE/100 g of DAP were selected, with some others with lower values added. This addition allowed us to check the correlation between the content of polyphenols determined chromatographically and in the method with the FC reagent. Chromatographic analyses showed the presence of 12 different polyphenols, of which 7 could be accurately determined using retention times and standards. 5 of them were partially identified based on specific absorption spectrum (Table S7 in Supplementary Materials). Among the analysed cultivars, the highest total content of polyphenols stood out in ‘Grochówka’, ‘Kantówka Gdańska’ and ‘James Grieve’ (4721.0, 4327.3 and 3898.6 mg/100 g of DAP, respectively) (Table S8 in Supplementary Materials). The lowest total content of polyphenols was found in ‘Grafsztynek Inflancki’ (only 478.7 mg/100 g of DAP), ‘Pepina Ribstona’ and ‘Książę Albert’ (826.1 and 854.6 mg/100 g of DAP respectively). Despite the smaller number of cultivars studied, the differences between them were close to 10 times—between 478.7 mg/100 g and 4721.0 mg/100 g. In extended (up to 67 cultivars) study, content of total polyphenols lay between 523.02 and 2723.96 mg/100 g DW [27]. Our analyses showed that the cultivars with a high content of polyphenols determined by the FC method do not always have a high content of total polyphenols determined by the HPLC method. For example, cultivars ‘Książę Albert’ and ‘Jakub Lebel’ have a TPC of 204.1 and 203.7 mg GAE/100 g of DAP, respectively, but only 854.6 and 1464.5 mg/100 g of DAP when analysed with the HPLC method. Compared with them, the cultivars ‘Grochówka’, ‘James Grieve’ and ‘Kantówka Gdańska’ are characterized by high values obtained from both methods. Despite the visible discrepancies, the rPearson correlation for the results of these two analyses is 0.61 (*p* ≤ 0.01), which proves a clear positive correlation. However, it should be remembered that the total content of polyphenols determined by the FC method is expressed in the mass equivalent of one compound—gallic acid—which is a relatively small molecule (170.1 g/M). In the case of the sum of masses of polyphenols determined by HPLC, we are dealing with compounds with different amounts of hydroxyl groups (which mainly determine the value of measurement by the FC method) and with much higher molecular masses (from 290.3 g/M to 610.5 g/M) than gallic acid. In addition, other non-polyphenol compounds may also affect the TPC results in the FC method.

Phenolic compounds content is one of the most intensely studied parameters and, depending on the analytical method used, the part of the fruit, the fresh or dry weight (DW) and, obviously, the apple cultivar and the cultivation agrotechnical method, the results vary in a very wide range [25]. For example, according to research [28], the content of chlorogenic acid in DAP stretches from 3 mg/100 g DW up to 233 mg/100 g DW (compared to 8–481 mg/100 g of DAP in our study). Similarly, for epicatechin, the content starts from 6 mg/100 g DW, and extends up to 349 mg/100 g DW (compared to 2–204 mg/100 g of DAP in our study). Our research explains that this approximately 100-fold difference in content is not a methodological error, but is instead a real significant variability in polyphenol levels, depending on the apple cultivars and other factors.

A significant number of cultivars and the results of analyses for various groups of compounds allowed us to determine certain correlations between them or the lack of a correlation (Table 1). The highest value for rPearson coefficient was 0.65, found for the total phenolic content and antioxidant properties, which gave a strong positive correlation. Phenolic compounds have long been recognized as the most valuable group of compounds, based on their activity as antioxidants [29]. Furthermore, in apples they are one of the most frequent radical scavenging compounds [30]. A strong correlation was also found between total polyphenol content and total phenolic acids content (0.64). This can be clearly attributed to the fact that simple phenolics are closely related with polyphenols metabolic pathways, biosynthesis or metabolism [31]. The correlation between total polyphenol content and total triterpenoic content was not so obvious, and to our knowledge has never been described before. The value of the rPearson coefficient 0.55 shows that levels of these two groups of compounds correlate quite clearly. On the other hand, a very low correlation was found between antioxidant properties and total phenolic acids content (0.13), which proves that small molecules of simple phenolic compounds are relatively weak scavengers of free radicals, in contrast to large and multifunctional polyphenols. The most conspicuous of all is the complete lack of correlation between triterpenoic acids content and antioxidant properties (−0.01). Other studies based on three apple cultivars show that the contribution of triterpenoids to the overall antioxidant potential is minor [15]. Now, we have shown that in an extended perspective of 30 cultivars there is no correlation between triterpenoids content and antioxidant potential.

The values of bioactive compounds for selected cultivars are presented in Table 2. For further analyses of cell cultures, two cultivars were selected. G and KG have the highest content of polyphenols determined by the most reliable method, i.e., HPLC. These two cultivars also have one of the highest contents of triterpenoic acids and TPC values. Some other cultivars had higher AP values; however, it should be remembered that carotenoids present in DAP could have had a significant impact on them.

### 3.2. Cytotoxic Activity of Extracts Obtained from KG and G Apple Peel

For further biological activity studies, two extracts were selected and obtained from the apple peels of cultivars richest in triterpenoids and phenolic compounds: Kantówka Gdańska (KG) and Grochówka (G). For the in vitro models, Caco-2 and HepG2 cell lines were chosen, which are often used in studies of biological activity of phytochemicals present in human diet [32,33]. Whereas Caco-2 cells are human immortalized intestinal epithelial cells used in bioavailability studies [34,35], the HepG2 cells are human immortalized liver-originated cells that are involved in hepatic carbohydrate and lipid metabolism [36].

**Figure 1:**
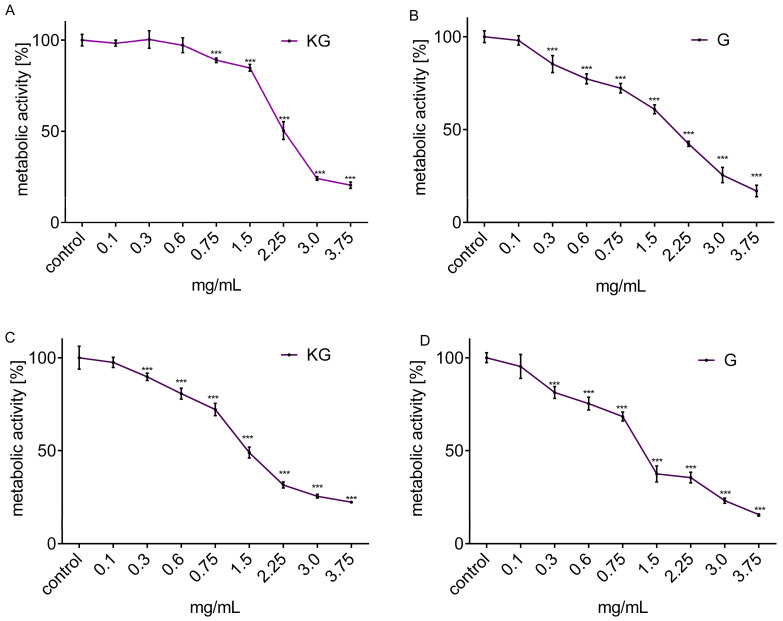
Metabolic activity of HepG2 (**A**,**B**) and Caco-2 (**C**,**D**) cells after 24 h incubation with extracts obtained from KG and G apple peel; metabolic activity was determined with MTT assay. The values represent the mean value ± SEM, n ≥ 8. Control cells were only exposed to the vehicle (medium). Statistical significance was calculated against the control cell culture with *** *p* ≤ 0.001.

First, the cytotoxic potential of the extracts against cells was determined for the concentration range between 0.1–3.75 mg/mL after 24 h of incubation (Figure 1A–D). Taking into account the type of cultivars, extract from KG was less cytotoxic than the extract from G. Table 3 presents the estimated values of the IC_50_ parameter, which means the concentration of the preparation that inhibits the metabolic activity of cells by 50%, in comparison to the control cells (not treated with DAP extracts). The relationship of cellular sensitivity based on IC_50_ was as follows: Caco-2 > HepG2. Considering the highest non-cytotoxic concentration (IC_0_) (Table 3), the unaffected metabolic activity of Caco-2 was observed after the cells’ incubation with 0.10 mg/mL in both extracts. In the case of HepG2 cells, they were more resistant to KG extract (0.60 mg/mL) than to G (0.10 mg/mL). To compare the direct effect of the preparations on cells present in the digestive tract, further experiments were performed on HepG2 and Caco-2 cells, and a concentration of 0.1 mg/mL (IC_0_) was selected for further studies.

**Figure 2:**
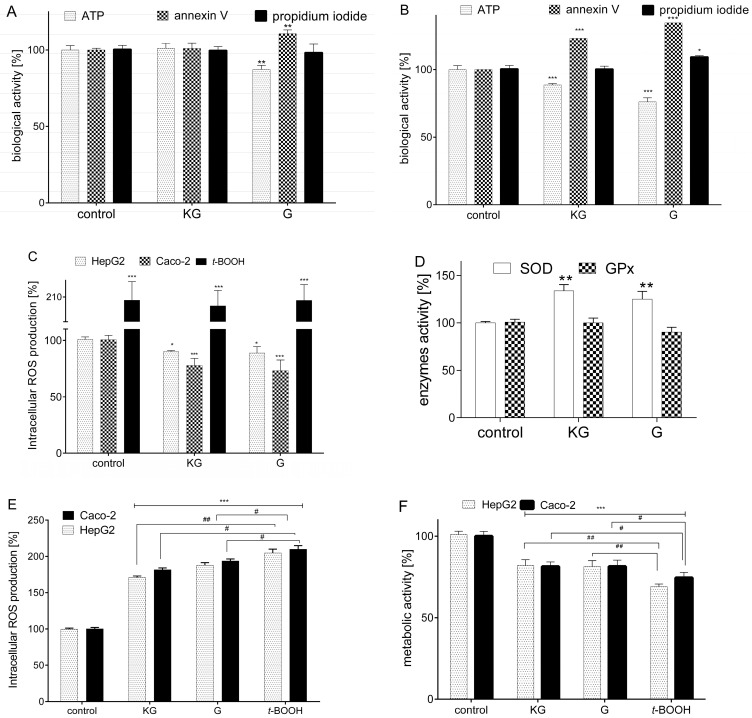

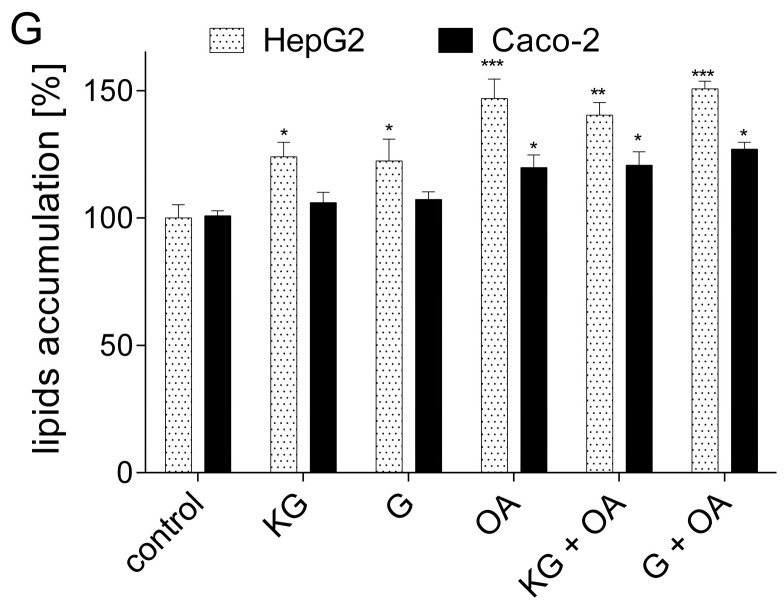
The effect of KG and G preparations at a concentration of 0.75 mg/mL after 24 h incubation on the intracellular level of ATP production, externalization of membrane phosphatidylserine (PS), and permeabilization of cell membrane in HepG2 (**A**) and Caco-2 (**B**) cells. The effect of extracts at a concentration 0.1 mg/mL on the intracellular level of ROS in HepG2 and Caco-2 cells; *t*-BOOH at a concentration of 500 µM was used as a positive control (**C**). The effect of extracts at a concentration of 0.1 mg/mL on the activity of SOD and GPx enzymes in Caco-2 cells (**D**). The cytoprotective effect of extracts at a concentration of 0.1 mg/mL on intracellular oxidative stress generation (**E**) and metabolic activity (**F**) after cells treatment with 500 µM *t*-BOOH for 2 h. The effect of extracts at a concentration of 0.1 mg/mL on lipid accumulation after 24 h incubation of HepG2 and Caco-2 cells in the presence of 300 µM oleic acid (OA) (**G**). Control cells were only exposed to the vehicle. The values in each column represent the mean ± SEM, n ≥ 4. Statistical significance was calculated against the control cell culture, with * *p* ≤ 0.05, ** *p* ≤ 0.01, *** *p* ≤ 0.001 or *t*-BOOH # *p* ≤ 0.05, ## *p* ≤ 0.01.

Since the difference between the IC_0_ and IC_50_ parameters does not exceed a 15-fold value of concentration (Table 1), in the next stage the effect of the extracts on the intracellular energy level and the mechanism of cytotoxicity was confirmed. The experimental data presented in Figure 2A confirmed the DAP extract from cultivar G at a concentration of 0.75 mg/mL, therefore not exceeding IC_50_, which reduced the intracellular level of ATP in HepG2 by almost 25%. A stronger effect of both extracts was observed in Caco-2 cells (Figure 2B), with a 15–25% decrease of ATP level (Figure 2B). This effect correlates with mitochondrial dysfunction and matches with a more significant inhibition of mitochondrial dehydrogenases activity (Figure 1A–D).

Irreversible reduction of intracellular ATP level triggers a programmed type of cell death, called apoptosis [37]. The early phase of apoptosis is manifested by the externalization of membrane phosphatidylserine (PS), the detection of which can be tracked using a fluorescent tag attached to annexin V having affinity for PS. It is known that the activation of apoptosis manifests itself by the activation of caspases that are responsible for the formation of condensed chromatin grains, DNA fragmentation and cell nucleus disintegration. Finally, apoptotic bodies are formed, containing the remains of a dying cell, which are in turn engulfed by neighbouring cells.

On the other hand, during the late phase of apoptosis and necrotic death, progressive permeabilization of cell membrane occurs, and this leads to increased water absorption by the cell, followed by its swelling and disintegration. The necrotic cell releases its contents into the external environment, including active enzymes and free radicals, which in turn promotes the generation of inflammation and carcinogenesis. Analysis of the results indicates that incubation of HepG2 cells with G increased the level of PS externalization by about 15% (Figure 2A). In the case of Caco-2 cells (Figure 2B), the same preparation, apart from inducing apoptosis (increasing the PS level by approx. 25%) also causes permeabilization of the cell membrane, enabling the intercalation of propidium iodide with nuclear DNA. This indicates the observation of a late phase of apoptosis or necrosis of the cells. Apoptosis in Caco-2 cells was also induced by the KG extract and in around 15% of externalizations of PS. Summing up, it can be assumed that the concentrations of the KG between IC_0_ and IC_50_, which have cytotoxic potential, induce an apoptotic type of cell death in HepG2 and Caco-2 cells. The observed permeabilization of the cell membrane in Caco-2 suggests that the G extract may induce necrotic or regulated necroptosis death, but this assumption requires further, more detailed, research.

### 3.3. Cytoprotective Effect of Extracts Obtained from KG and G Apple Peel Extracts

Oxygen free radicals (ROS) in the cellular system play an important role as signalling substances; however, their elevation promotes cell damage and death via oxidation of proteins, lipids and DNA. Cells of the digestive system, due to the metabolic changes of compounds supplied with food (including metabolites generated by the intestinal microbiota), are particularly exposed to the toxic effects of free radicals. Therefore, in the next step, the antioxidant effect of preparations at IC_0_ concentration in Caco-2 and HepG2 cells was analysed. Antioxidant activity was tested using the H_2_DCFDA marker, which emits fluorescence in the oxidized form of DCF due to the action of radicals. The known chemical inducer of oxidative stress, 500 µM *tert*-butylhydroperoxide (*t*-BOOH) was used as a positive control, and increased the intracellular ROS level more than 200%, in comparison to control cells (Figure 2C). A higher potential for reducing free radicals was shown for the G extract, which reduced oxidative stress in cells by approx. 10–25%. The extract from KG had a weaker effect, decreasing ROS level by almost 5%. A stronger effect in ROS reduction was observed for Caco-2 cells than for HepG2. Reducing the level of ROS by phenolic compounds can result from the direct chemical reaction; however, they may also affect the activity and level of cellular enzymes involved in the regulation of redox-sensitive signalling pathways, such as sodium dismutase (SOD) and glutathione peroxidase (GPx) [38]. Since a stronger effect of preparations on ROS reduction was observed for Caco-2 cells, we checked this enzymes activity in enterocytes. As presented in Figure 2D, both extracts elevated the activity of SOD by about 20–25% and had no effect on GPx.

The observed ability of the preparations to reduce the intracellular level of ROS allowed us to determine their cytoprotective potential in cells with induced chronic oxidative stress. In vivo, increased oxidative stress is found in metabolic diseases such as type 2 diabetes or obesity and is generated in the presence of elevated glucose or free fatty acid concentrations [39]. Here, a chemical inducer of oxidative stress was used: 500 µM *t*-BOOH. As shown in Figure 2E, cell preincubation with extracts at IC_0_ = 0.1 mg/mL for 24 h reduced the oxidative stress induced by *t*-BOOH by 15–25%, in comparison to cells treated with only *t*-BOOH. The cytoprotective effect of KG was about 10% stronger than that of G. Nonetheless, both preparations were more effective as cytoprotective agents in HepG2 cells. On the other hand, *t*-BOOH reduced the metabolic activity of the cells to 70–75% (Figure 2F). Pre-incubation of cells with extracts increased their metabolic activity by about 10%, compared to cells treated with *t*-BOOH alone. As previously, the protective effect of the extracts was stronger by almost 5% in HepG2 cells than in Caco-2 cells.

It is not just ROS that damages cellular structures, since disturbance of cell activity can also be caused by lipotoxicity. This effect may lead to steatosis of cells from peripheral tissue, which are not adapted to the accumulation of lipids, which in turn impairs their functioning by generating an inflammatory response, impaired glucose uptake and insulin resistance [40]. Therefore, in the next step the effect of preparations on lipid accumulation in the presence of 300 µM oleic acid was measured. For this purpose, the Nile red probe was used, which emits fluorescence in a lipophilic environment. As seen in Figure 2G, treatment of cells with oleic acid induced greater lipid accumulation in HepG2 cells (nearly 45% compared to control cells), whereas in Caco-2 cells the fluorescence level increased by about 15%. Both extracts did not protect HepG2 cells against steatosis during co-incubation with oleic acid and even increased the level of lipid accumulation in cells by about 20%. In the case of Caco-2 cells, the effect of stimulating steatosis was not observed in a statistically significant way, but, similar to hepatocytes, the preparations did not protect the cells against lipid accumulation stimulated by oleic acid. In the latter case, the lipid level was increased by about 15%, compared to untreated cells. Similar observation of increasing lipid accumulation by cells was described for extracts obtained from Jonagold apple peel, which contained phenolic compounds and triterpenoids [41].

## 4. Conclusions

Within 30 old apple cultivars studied, significant differences were shown in AP, TPC, phenolic acids, polyphenols and triterpenoid levels. The highest values for AP were found in ‘Książę Albrecht’, and for TPC in ‘James Grieve’; however, based on more accurate chromatographic methods, we obtained results that show the highest concentration of sum of polyphenols in G and KG. These two cultivars also have the highest concentrations of triterpenoids. Based on these findings, cell line-based in vitro studies with G and KG DAP extracts have been conducted. The studies demonstrated that the extract from KG was less cytotoxic than the extract from G in both studied cell lines. KG concentration between IC_0_ and IC_50_ induced apoptotic type of cell death in studied cells, whereas G extract might induce necrosis. A higher potential for reducing free radicals was shown for the G extract, which reduced oxidative stress in cells by up to 25%. Both extracts reduced the oxidative stress induced by *t*-BOOH by 15–25%. Extracts from dried apple peels of both cultivars did not protect the studied cells against lipid accumulation stimulated by oleic acid. Based on these studies, it can be concluded that the pro-health potential of apples’ phytocompounds relies mostly on their antioxidant potential.

As revealed in our analyses, the contents of individual groups of bioactive compounds differ by up to two orders of magnitude between varieties. These compounds have strong health-promoting properties and their natural supplementation via raw fruits present in everyday diet will have a strong impact on the counteraction of lifestyle-induced diseases. Therefore, the results of this study support promoting fruit varieties rich in bioactive compounds in the market to improve consumer health.

## Figures and Tables

**Table 1:** Correlation matrix between each group of analysed compounds.

	Antioxidant Properties (DPPH Method)	Total Phenolic Content (FC Method)	Total Phenolic Acids Content (GC–MS Method)	Total Triterpenoic Content (GC–MS Method)	Total Polyphenol Content (HPLC–DAD Method)
Antioxidant properties (DPPH method)	X	0.65	0.13	−0.01	0.30
Total phenolic content (FC method)	0.65	X	0.32	0.38	0.61
Total phenolic acids content (GC-MS method)	0.13	0.32	X	0.28	0.64
Total triterpenoic content (GC-MS method)	−0.01	0.38	0.28	X	0.55
Total polyphenol content (HPLC-DAD method)	0.30	0.61	0.64	0.55	X

**Table 2:** Antioxidant properties, total phenolic content, total phenolic acids content, triterpenoic content and polyphenol content in selected cultivars. Bold font signifies values higher than average from all cultivars in each analysis. Data represent the mean ± SD. Different letters correspond to statistically significant differences between cultivars, according to one-way ANOVA followed by Tukey post hoc test (*p* < 0.05).

Cultivar	Antioxidant Properties [mgTE/100 g of DAP]	Total Phenolic Content [mg GAE/100 g of DAP]	Total Phenolic Acids Content [mg/100 g of DAP]	Total Triterpenoic Content [mg/100 g of DAP]	Total Polyphenols Content [mg/100 g of DAP]
Boskoop	852.1 ± 45.5 ^c^	164.6 ± 6.8 ^a^	**870.1 ± 25.4 ^ghij^**	**1494.1 ± 50.7 ^fgh^**	**2225.3 ± 79.2 ^ef^**
Grochówka	**1105.5 ± 51.8 ^fghij^**	**217.0 ± 10.4 ^ghij^**	**872.1 ± 26.1 ^hij^**	**1976.8 ± 48.5 ^k^**	**4721.0 ± 99.3 ^k^**
Jakub Lebel	**1129.8 ± 53.2 ^ijk^**	**203.7 ± 10.6 ^defg^**	401.3 ± 18.4 ^d^	**1491.8 ± 49.6 ^fgh^**	1464.5 ± 46.6 ^b^
James Grieve	**1090.2 ± 46.7 ^ghij^**	**246.6 ± 12.4 ^k^**	**685.4 ± 29.9 ^f^**	**1759.7 ± 40.2 ^j^**	**3898.6 ± 87.1 ^i^**
Kantówka Gdańska	**951.0 ± 21.3 ^de^**	**214.2 ± 2.5 ^ghi^**	**1661.0 ± 35.6 ^k^**	**1617.7 ± 42.9 ^i^**	**4327.3 ± 110.2 ^j^**
Kronselska	536.9 ± 32.2 ^a^	**199.4 ± 5.3 ^cdef^**	**627.4 ± 10.7 ^e^**	**1518.5 ± 38.3 ^fgh^**	**2976.0 ± 67.5 ^gh^**
Książę Albert	**1058.8 ± 71.0 ^efg^**	**204.1 ± 15.7 ^efgh^**	210.4 ± 14.3 ^a^	983.0 ± 22.0 ^a^	854.6 ± 23.9 ^a^
Książę Albrecht Pruski	**1136.5 ± 46.4 ^ijk^**	**199.1 ± 7.3 ^cde^**	269.4 ± 5.8 ^b^	**1297.6 ± 29.1 ^cde^**	**3039.3 ± 67.4 ^gh^**
Niezrównane Peasgooda	647.5 ± 39.6 ^b^	**176.7 ± 5.6 ^b^**	**853.6 ± 30.3 ^ghi^**	**1304.7 ± 36.8 ^cde^**	1846.8 ± 56.0 ^cd^
Schieblers Taubenapfel	**1076.6 ± 36.8 ^fgh^**	**193.7 ± 27.1 ^c^**	310.0 ± 17.1 ^c^	1063.1 ± 34.5 ^b^	**2275.0 ± 47.1 ^ef^**
Złota Reneta	**1006.5 ± 61.8 ^ef^**	**195.5 ± 8.1 ^cd^**	**801.3 ± 30.8 ^gh^**	**1274.3 ± 31.9 ^cde^**	1858.5 ± 21.9 ^cd^

**Table 3:** Comparison of IC_50_ and IC_0_ parameters of extracts obtained from ‘Kantówka Gdańska’ and ‘Grochówka’ apple peel extracts.

Cell Line	IC_50_ [mg/mL]		IC_0_ [mg/mL]	
	KG	G	KG	G
HepG2	2.25	2.00	0.60	0.10
Caco-2	1.50	1.25	0.10	0.10

## Data Availability

The original contributions presented in the study are included in the article, further inquiries can be directed to the corresponding author.

## Supplementary Materials

The following supporting information can be downloaded at: https://www.mdpi.com/article/10.3390/foods13071014/s1, Table S1: Old apple cultivars, the fruit of which were analyzed in this paper; Table S2: Average antioxidant properties and total phenolic content in all analyzed cultivars; Table S3: Phenolic acids identification; Table S4: Comparison of phenolic acids content in analyzed apple cultivars; Table S5: Triterpenoids identification; Table S6: Comparison of triterpenoic acids and their derivatives content in analyzed apple cultivars; Table S7: Polyphenols identification; Table S8: Comparison of polyphenols content in analyzed apple cultivars.
