# Supplementary material for: Old Apple Cultivars as a Natural Source of Phenolics and Triterpenoids with Cytoprotective Activity on Caco-2 and HepG2 Cells
Source: Foods. 2024 Mar 26;13(7):1014. doi: 10.3390/foods13071014 (PMC11011742; doi:10.3390/foods13071014)
Supplement: Supplementary file 1 [file foods-13-01014-s001.zip › foods-2913529-supplementary.pdf]

Table S1. Old apple cultivars, the fruit of which were analyzed in this paper. [*Atlas dawnych odmian jabłoni ISBN 978-83-61821-47-2; The New Book of Apples ISBN 0-09-188398-9; Directory of Apple Cultivars ISBN 1-874275-40-8*]

Cultivar name	Fruit appearance	Other names	Region and year of origin	Additional information
Boskoop	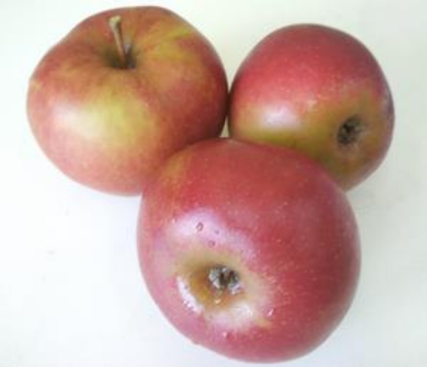	Belle de Boskoop, Schone van Boskoop	Netherlands, 1856	crossbreeding of 'McIntosh' and 'Ben Davies'
Dean's Codlin	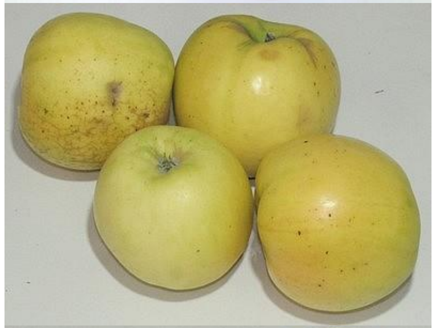	Deans Küchenapfel	England, 1870	also known as 'Potts' Seedling'
Galloway Pippin	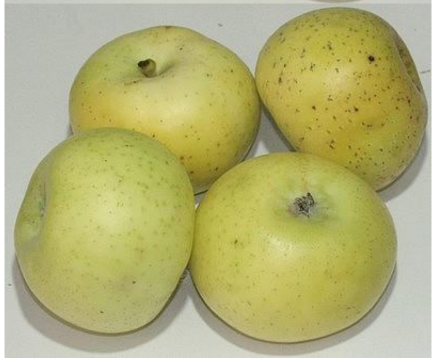		south-west Scotland, 1871	-

Grafsztynek Inflancki	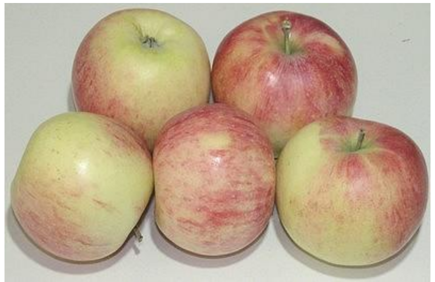	Gravenstein	Latvia/Estonia, XVII	originated from a German cultivar 'Grafsztynek Prawdziwy'
Grochówka	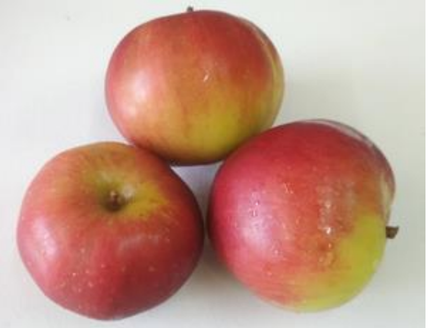		Germany, after 1750	the name comes from the fruit's high resistance to storage
Jakub Lebel	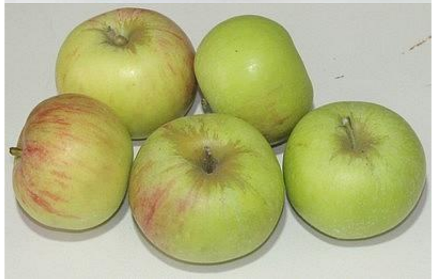	Jacob Lebel, Jacques Lebel	France, ca. 1825	-
James Grieve	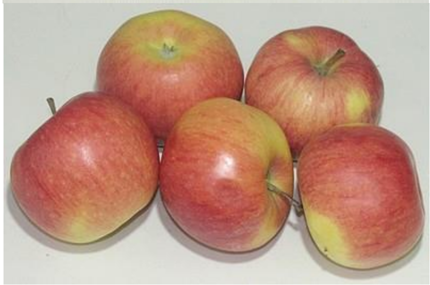		Scotland, ca. 1890	grown from seedlings of cultivar 'Potts Seedling'

Kalwila Aderslebeńska	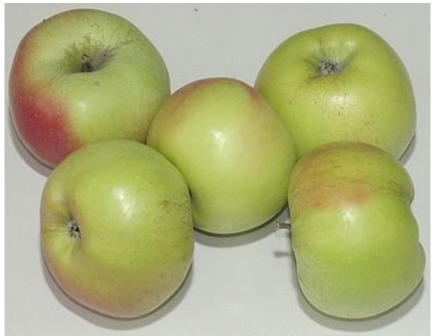	Germany, 1839	crossbreeding of 'Kalwila Biała Zimowa' and 'Grafsztynek Prawdziwy'
Kantówka Gdańska	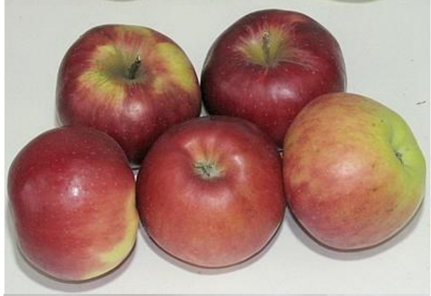	Netherlands, ca. 1760	the cultivar originated probably in the vicinity of Gdańsk city
Koksa Pomarańczowa	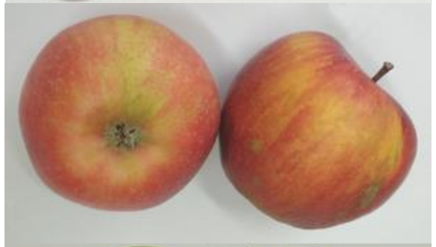	England, 1858	seedling of 'Pepina Ribstona'
Kosztela	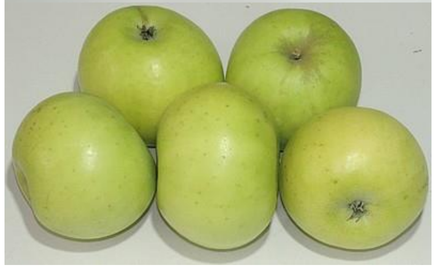	Poland, XVII	one of the oldest European cultivars

Kronselska	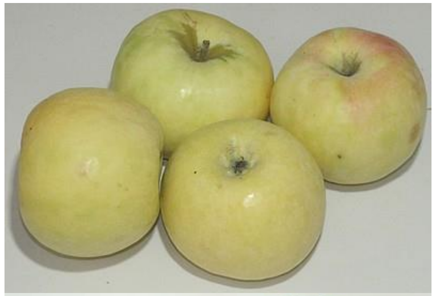	Transparente de Croncels	France, 1869	seedling of 'Antonówka Zwykła'
Krótkonóżka Królewska	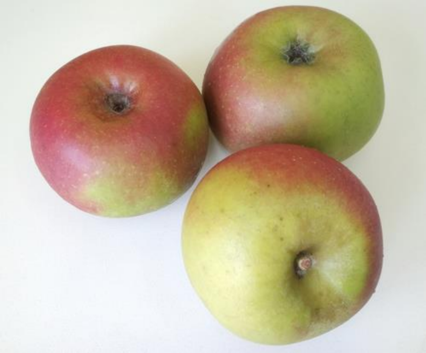		probably Netherlands, the end of the XVIth	one of the oldest European cultivars
Książę Albert	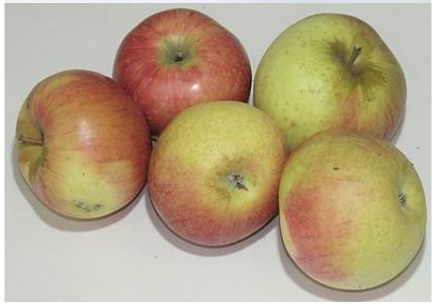	Lane's Prince Albert	England, 1840	crossbreeding of 'Russet Nonpareil' and 'Wellington'

Książę Albrecht Pruski	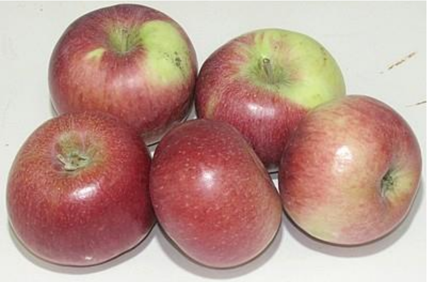	Prince Albrecht of Prussia	Germany, 1865	crossbreeding of 'Aporta' and 'Reneta Baumanna'
Malinowa Oberlandzka	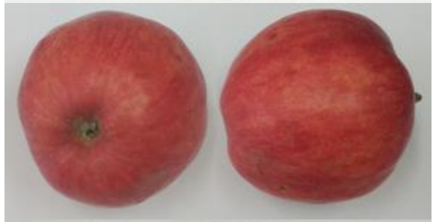	Raspberry Oberland	Germany, 1854	characteristically pink-colored flesh
Niezrównane Peasgooda	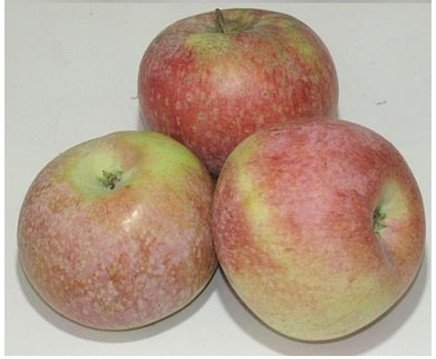	Peasgood's Nonsuch	England, ca. 1858	seedling found in the Royal Gardens in London

Pepina Linneusza	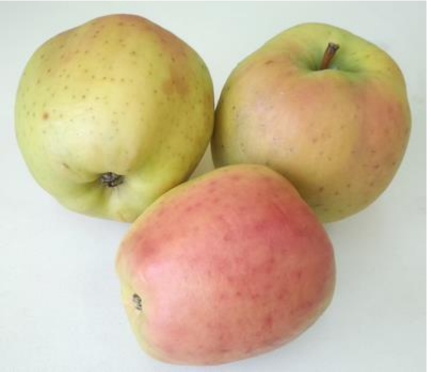	Linneus' Pippin	USA, 1750	known by many names, but the most original is 'Coxe'
Pepina Ribstona	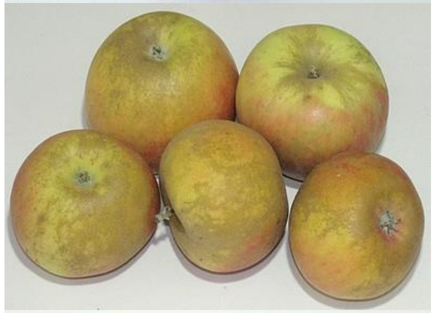	Ribston Pippin	England, the end of the XVIIth	
Piękna z Rept	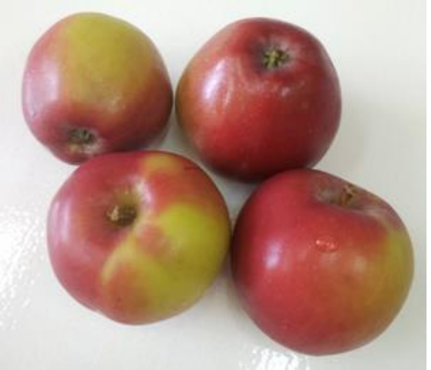	Beauty of Repty	Poland, time unknown	bred in the Palace Garden in Repty (nowadays a district of Tarnowskie Góry); characteristic, very hard flesh

Reneta Blenheimska	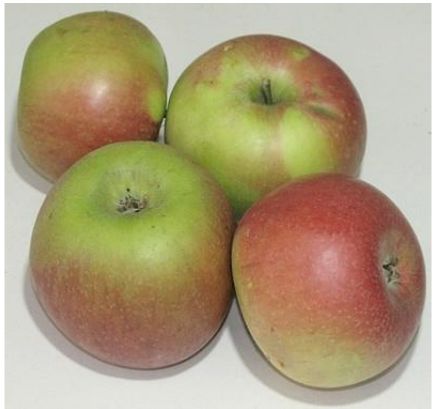	Blenheim Orange, Reinette de Blenheim	England, ca. 1740	-
Reneta Harberta	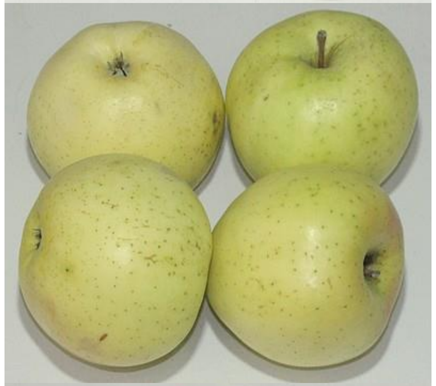	Harbert's Reinette, Harbert's Apfel	Germany, ca. 1820	-
Reneta Kanadyjska	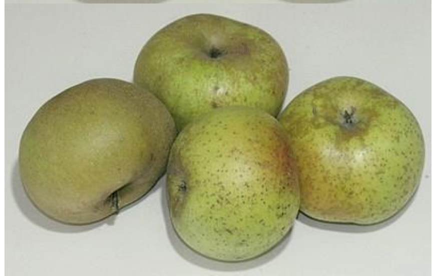	Canadian Reinette, Reinette du Canada	France, 1771	originated from France, then it was sent to Canada, from where it returned to Europe under the name of 'Reneta Kanadyjska'

Reneta Kulona	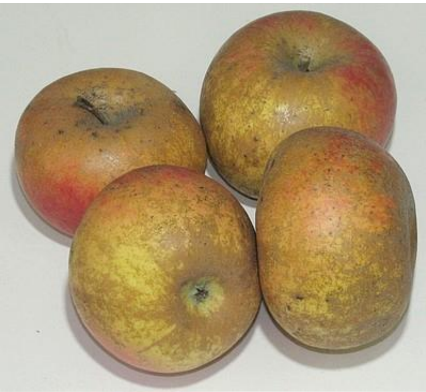	Coulon`s Reinette, Reinette Coulon	Belgium, 1856	-
Reneta Strauwalda	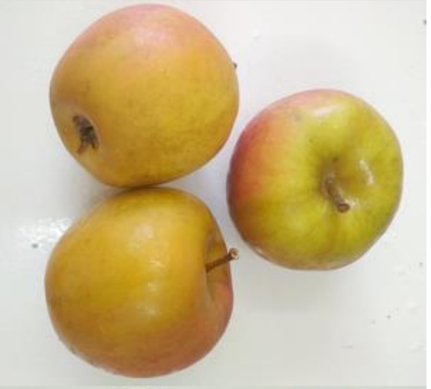	Strauwald's Reinette, Strauwalds Goldparmane	Germany, 1856	-
Reneta z Brownlee	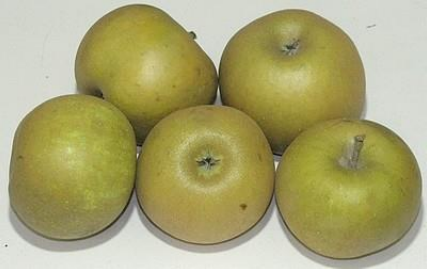	Brownlees' Russet	England, ca. 1840	-

Schieblers Taubenapfel	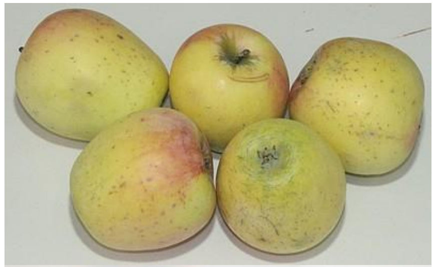		Germany, 1st half of the XVIIIth	-
Szara Reneta	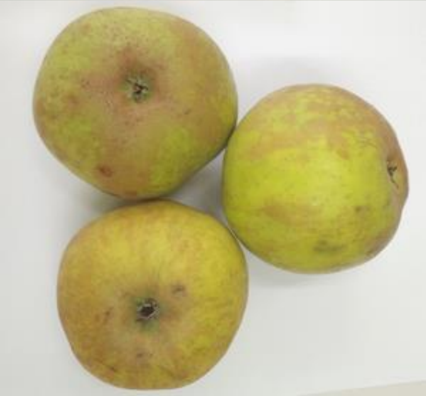	Grey Reinette	France, ca. 1650	one of the oldest European cultivars
Złota Reneta	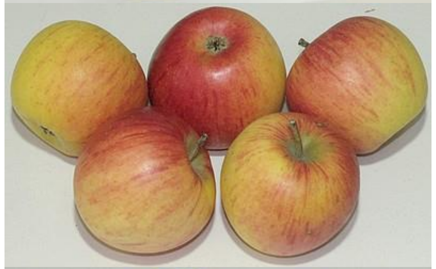	Golden Reinette	France, ca. 1510	one of the oldest European cultivars
Złotka Kwidzyńska	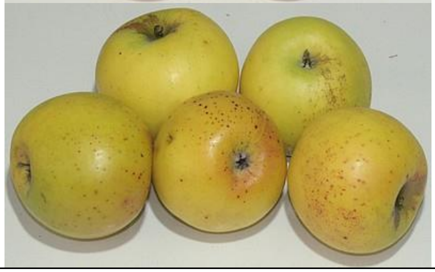		Poland, time unknown	cultivar found in the village of Nieborów near the town of Kwidzyn

Table S2. Average antioxidant properties and total phenolic content in all analyzed cultivars. Antioxidant properties are presented as the equivalent of Trolox [mg TE/100g of DAP] and total phenolic content as the equivalent of gallic acid [mg GAE/100g of DAP]. Values are presented as the average of all analyzed samples within the variant ± standard deviation. One-way ANOVA analyses shows statistically significant differences between cultivars ($P < 0.05$).

Cultivar	Antioxidant properties [mg TE/100g of DAP]	Total phenolic content [mg GAE/100g of DAP]
Boskoop	852.1 ± 45.5	164.6 ± 6.8
Deans' Codlin	833.3 ± 41.7	158.2 ± 9.3
Galloway Pippin	716.4 ± 43.8	144.7 ± 7.7
Grafsztynek Inflancki	481.0 ± 33.2	143.2 ± 11.2
Grochówka	1105.5 ± 51.8	217.0 ± 10.4
Jakub Lebel	1129.8 ± 53.2	203.7 ± 10.6
James Grieve	1090.2 ± 46.7	246.6 ± 12.4
Kalwila Aderslebeńska	999.8 ± 44.3	174.6 ± 9.9
Kantówka Gdańska	951.0 ± 21.3	214.2 ± 2.5
Koksa Pomarańczowa	639.7 ± 35.4	169.9 ± 10.1
Kosztela	411.1 ± 46.1	161.4 ± 9.5
Kronselska	536.9 ± 32.2	199.4 ± 5.3
Krótkonóżka Królewska	1084.3 ± 42.9	198.4 ± 1.6
Książę Albert	1058.8 ± 71.0	204.1 ± 15.7
Książę Albrecht Pruski	1136.5 ± 46.4	199.1 ± 7.3
Malinowa Oberlandzka	612.2 ± 23.5	137.4 ± 9.0
Niezrównane Peasgooda	647.5 ± 39.6	176.7 ± 5.6
Pepina Linneusza	767.4 ± 15.8	158.0 ± 34.6
Pepina Ribstona	708.7 ± 29.8	147.4 ± 14.1
Piękna z Rept	807.6 ± 12.6	155.9 ± 9.8
Reneta Blenheimska	1152.9 ± 73.7	185.9 ± 23.3
Reneta Harberta	813.0 ± 26.5	159.4 ± 17.3
Reneta Kanadyjska	1004.3 ± 51.4	188.2 ± 8.7
Reneta Kulona	1079.4 ± 33.7	173.6 ± 11.5
Reneta Strauwalda	1107.5 ± 46.5	186.6 ± 10.9
Reneta z Brownlee	755.1 ± 25.7	145.4 ± 14.8
Schieblers Taubenapfel	1076.6 ± 36.8	193.7 ± 27.1
Szara Reneta	1061.0 ± 39.2	178.0 ± 16.2
Złota Reneta	1006.5 ± 61.8	195.5 ± 8.1
Złotka Kwidzyńska	992.1 ± 56.3	194.3 ± 4.4

Table S3. Phenolic acids identification. Abbreviations: RI_{exp}, retention index of TMS derivative of the compound in samples on BPX-5 column; RI_S, retention index of TMS derivative of standard on BPX-5 column; RI_{lit}, retention index of TMS derivative reported in literature on BPX-5 or similar column; MS, identification based on available libraries spectra; S, identification based on standard; nd, no data; *cinnamic acid was not present in extracts from DAP but was used as standard to quantification.

**Compound**	**RI_{exp}**	**RI_S**	**RI_{lit}**	**Identification**	**Quantity evaluated using standard**
Cinnamic acid*		1541	1543	MS/S	
4-Hydroxybenzoic acid	1635		1637	MS	Gallic acid
NI	1715				Gallic acid
Phloretic acid	1750		1750	MS	Cinnamic acid
3,4-Dihydroxybenzoic acid	1801		1800	MS	Gallic acid
p-Coumaric acid	1956		1955	MS	Cinnamic acid
Gallic acid	1988	1988	1986	MS/S	Gallic acid
Ferulic acid	2108		2106	MS	Cinnamic acid
Caffeic acid	2161	2161	2158	MS/S	Caffeic acid

Table S4. Comparison of phenolic acids content in analyzed apple cultivars. Values in mg/100 g of DAP. Values presented as the average of all analyzed samples within the variant ± standard deviation. One-way ANOVA analyses shows statistically significant differences between cultivars (P < 0.05). Abbreviations: <LOQ, detected but values below the limit of quantification; nd, compound not detected.

**Cultivar**	4-Hydroxy-benzoic acid	NI	Phloretic acid	3,4-dihydroxy-benzoic acid	*p*-Coumaric acid	Gallic acid	Ferulic acid	Caffeic acid	**Total content:**
Boskoop	32.70 ± 4.33	244.04 ± 20.94	63.86 ± 3.55	29.59 ± 1.07	37.77 ± 0.25	377.96 ± 45.30	18.68 ± 0.32	65.53 ± 2.85	870.13 ± 25.44
Galoway Pippin	151.96 ± 14.12	118.61 ± 22.62	61.96 ± 2.23	16.23 ± 0.81	66.01 ± 0.32	67.64 ± 6.20	54.11 ± 1.20	93.15 ± 1.79	629.67 ± 15.84
Grafsztynek Inflancki	119.08 ± 9.35	91.42 ± 22.06	nd	nd	46.83 ± 1.25	58.12 ± 5.47	18.96 ± 0.22	42.28 ± 0.73	376.68 ± 13.89
Grochówka	172.47 ± 16.40	nd	100.11 ± 5.26	54.98 ± 2.67	170.95 ± 3.27	280.09 ± 10.79	nd	93.45 ± 1.06	872.05 ± 26.11
Jakub Lebel	nd	64.14 ± 9.36	63.82 ± 5.92	15.45 ± 0.73	71.65 ± 5.23	117.63 ± 9.56	nd	68.63 ± 2.30	401.32 ± 18.44
James Grieve	75.55 ± 15.53	62.60 ± 10.08	44.42 ± 1.63	<LOQ	60.15 ± 2.09	96.90 ± 7.34	26.76 ± 0.32	319.01 ± 4.53	685.40 ± 29.87
Kalwila Aderslebeńska	nd	48.78 ± 7.65	127.69 ± 4.09	14.21 ± 0.25	61.59 ± 0.37	120.95 ± 11.07	nd	43.88 ± 0.43	417.10 ± 14.97
Kantówka Gdańska	50.74 ± 7.87	269.36 ± 23.34	240.59 ± 5.20	140.09 ± 3.56	61.43 ± 3.04	223.04 ± 12.40	402.64 ± 2.56	273.15 ± 3.51	1661.03 ± 35.61
Koksa Pomarańczowa	39.36 ± 10.11	129.34 ± 10.85	133.71 ± 2.19	30.64 ± 1.11	67.26 ± 3.58	127.57 ± 9.65	53.46 ± 0.73	54.20 ± 1.01	635.53 ± 21.50
Kosztela	99.52 ± 12.64	39.60 ± 8.21	nd	nd	34.23 ± 1.35	<LOQ	nd	36.26 ± 0.67	209.60 ± 13.47
Kronselska	71.58 ± 11.76	62.65 ± 7.63	111.23 ± 2.13	nd	137.51 ± 4.38	106.91 ± 8.88	33.22 ± 1.01	104.27 ± 0.97	627.38 ± 10.65
Krótkonóżka Królewska	<LOQ	368.31 ± 12.57	160.70 ± 1.38	74.63 ± 0.49	41.40 ± 1.30	147.47 ±10.10	211.88 ± 2.13	149.29 ± 0.52	1153.67 ± 30.20
Książę Albert	80.87 ±7.35	33.22 ± 5.76	nd	nd	28.52 ± 0.33	67.80 ± 7.15	nd	<LOQ	210.41 ± 14.28
Książę Albrecht Pruski	81.64 ±9.70	27.04 ±9.91	41.51 ± 0.69	nd	32.59 ± 0.77	86.63 ± 11.14	nd	nd	269.41 ± 5.75
Malinowa Oberlandzka	nd	79.66 ± 0.98	<LOQ	nd	19.00 ± 0.90	<LOQ	35.13 ± 0.11	88.05 ± 0.40	221.86 ± 3.75
Niezrównane Peasgooda	89.64 ± 12.34	259.92 ± 15.86	220.55 ± 9.49	nd	54.32 ± 3.79	18.67 ± 2.07	110.32 ± 0.77	100.20 ± 1.21	853.63 ± 30.26
Pepina Linneusza	103.03 ± 9.33	126.00 ± 12.08	52.97 ± 0.75	27.98 ± 1.22	48.75 ± 0.23	183.60 ± 9.42	46.13 ± 0.36	52.46 ± 0.49	640.91 ± 25.67
Pepina Ribstona	144.07 ±10.14	31.12 ± 7.32	64.37 ± 0.89	nd	41.21 ± 0.63	<LOQ	nd	<LOQ	280.77 ± 21.44
Piękna z Rept	92.98 ± 7.40	34.03 ± 6.98	120.12 ± 2.89	42.98 ± 1.89	48.18 ± 0.74	258.03 ± 14.51	nd	32.20 ± 0.60	628.51 ± 14.61
Reneta Blenheimska	nd	62.25 ± 10.44	77.28 ± 3.09	31.03± 2.63	77.67 ± 2.35	129.35 ± 8.74	29.37 ± 0.88	44.20 ± 1.58	451.15 ± 11.12

Reneta Harberta	96.82 ± 11.56	119.17 ± 11.03	63.91 ± 3.47	24.86 ± 1.04	24.29 ± 0.68	117.54 ± 7.11	50.34 ± 0.90	46.73 ± 2.75	543.64 ± 15.14
Reneta Kandyjska	87.25 ±9.98	67.74 ± 10.94	125.68 ± 4.99	26.51 ± 0.42	67.17 ± 1.49	87.13 ± 6.35	27.56 ± 0.06	52.83 ± 1.29	541.86 ± 10.62
Reneta Kulona	42.07 ± 4.04	74.54 ± 12.77	128.82 ± 4.99	37.88 ± 3.00	87.61 ± 0.98	245.95 ± 17.41	37.42 ± 0.22	81.07 ± 3.68	735.36 ± 30.45
Reneta z Brownlee	143.48 ± 11.10	33.06 ± 2.93	147.03 ± 1.64	35.35 ± 4.26	109.94 ± 5.13	129.64 ± 14.72	52.87 ± 0.74	66.19 ± 2.02	717.56 ± 24.39
Schieblers Taubenapfel	54.12 ± 6.63	nd	82.61 ± 2.73	20.41 ± 1.01	20.41 ± 0.93	113.42 ± 9.10	19.06 ± 0.63	nd	310.04 ± 17.14
Szara Reneta	63.39 ± 1.27	nd	135.83 ± 5.43	10.99 ± 0.11	22.34 ± 1.31	<LOQ	11.85 ± 0.71	20.31 ± 0.81	264.71 ± 16.92
Złota Reneta	63.53 ± 2.25	101.50 ± 8.99	232.18 ± 11.96	131.87 ± 3.91	78.66 ± 0.48	97.39 ± 8.35	66.97 ± 0.64	29.25 ± 0.25	801.34 ± 30.80

Table S5. Triterpenoids identification. Abbreviations: RT_{exp}, retention time of compound in samples on BPX-5 column; RT_S, retention time of standard on BPX-5 column; MS, identification based on available libraries spectra; S, identification based on standard.

Compound	RT_{exp}	RT_S	Identification	Quantity evaluated using standard
α-Amyrin	11.88		MS	Betulin
Betulin	13.83	13.87	MS/S	Betulin
Oleanolic acid	14.08	14.08	MS/S	Oleanolic acid
Betulinic acid	14.37	14.38	MS/S	Betulinic acid
Ursolic acid	15.07	15.08	MS/S	Ursolic acid
Maslinic acid	16.28		MS	Oleanolic acid
Corosolic acid	17.05		MS	Ursolic acid

Table S6. Comparison of triterpenoic acids and their derivatives content in analyzed apple cultivars. Values in mg/100 g of DAP. NI - not identified compound; E/U - erythrodiol or uvaol (lack of standard to identify, MS spectrum inconclusive). One-way ANOVA analyses shows statistically significant differences between cultivars (P < 0.05). Abbreviations: <LOQ, detected but values below the limit of quantification; nd, compound not detected.

**Cultivar**	α-Amyrin	NI	E/U	Betulin	Oleanolic acid	Betulinic acid	Ursolic acid	Maslinic acid	Corosolic acid	**Total content:**
Boskoop	nd	nd	12.1 ± 1.2	nd	295.3 ± 17.8	12.7 ± 1.3	898.1 ± 41.2	42.4 ± 0.9	233.5 ± 10.3	1494.1 ± 50.7
Deans' Codlin	nd	nd	nd	nd	276.8 ± 18.7	27.2 ± 3.0	871.0 ± 39.1	55.7 ± 0.8	149.9 ± 10.1	1380.6 ± 47.1
Galowany Pipping	nd	nd	9.0 ± 0.8	nd	134.1 ± 9.9	11.0 ± 0.8	505.5 ± 22.7	33.2 ± 1.1	39.2 ± 5.2	732.0 ± 35.2
Grafsztynek Inflancki	nd	nd	13.6 ± 1.0	nd	199.8 ± 22.1	17.3 ± 1.6	901.7 ± 47.0	15.4 ± 0.0	97.2 ± 7.1	1245.0 ± 33.8
Grochówka	56.8 ± 4.4	nd	36.1 ± 2.4	12.7 ± 0.3	361.0 ± 27.3	5.4 ± 0.0	1401.1 ± 60.3	<LOQ	103.7 ± 9.4	1976.8 ± 48.5
Jakub Lebel	nd	nd	nd	nd	229.9 ± 23.5	21.5 ± 2.2	1007.4 ± 52.4	56.2 ± 1.2	176.8 ± 11.5	1491.8 ± 49.6
James Grieve	nd	nd	nd	nd	283.3 ± 30.2	28.8 ± 1.8	1170.7 ± 55.6	68.5 ± 1.4	208.4 ± 15.9	1759.7 ± 40.2
Kalwila Aderslebeńska	nd	nd	14.8 ± 2.2	nd	213.5 ± 18.6	18.4 ± 2.1	1020.0 ± 49.4	20.1 ± 1.0	100.8 ± 6.8	1387.6 ± 31.5
Kantówka Gdańska	nd	nd	7.0 ± 0.1	9.3 ± 0.4	238.1 ± 14.0	18.1 ± 1.4	994.2 ± 48.7	101.6 ± 2.5	249.4 ±14.0	1617.7 ± 42.9
Koksa Pomarańczowa	nd	nd	<LOQ	nd	272.1 ± 24.1	28.0 ± 2.7	744.6 ± 32.5	67.1 ± 1.3	172.2 ±12.4	1284.0 ± 31.0
Kosztela	nd	nd	nd	nd	155.0 ± 12.2	16.0 ± 1.5	854.9 ± 33.0	80.2 ± 1.7	61.7 ± 5.7	1167.8 ± 29.2
Kronselska	nd	nd	10.3 ± 0.2	nd	255.7 ± 26.9	17.0 ± 1.1	1072.6 ± 45.1	24.2 ± 0.5	138.7 ± 12.1	1518.5 ± 38.3
Krótkonóżka Królewska	nd	nd	<LOQ	nd	277.1 ± 15.4	22.2 ± 1.9	977.3 ± 53.0	142.8 ± 2.4	332.1 ± 19.9	1751.5 ± 34.9
Książę Albert	nd	nd	45.5 ± 4.5	nd	163.1 ± 14.3	14.8 ± 1.5	503.2 ± 21.2	68.7 ± 0.7	187.7 ± 17.3	983.0 ± 22.0
Książę Albrecht Pruski	nd	nd	29.4 ± 3.1	nd	154.0 ± 11.0	3.7 ± 0.5	862.3 ± 30.8	57.6 ± 0.7	190.6 ± 15.7	1297.6 ± 29.1
Malinowa Oberlandzka	nd	19.4 ±2.1	34.5 ± 2.3	<LOQ	257.1 ± 23.7	30.5 ± 2.7	1065.5 ± 44.3	55.7 ± 0.6	237.0 ± 16.8	1699.7 ± 43.5
Niezrównane Peasgooda	nd	nd	33.0 ± 0.7	nd	181.6 ± 17.6	<LOQ	902.2 ± 40.9	54.0 ± 0.7	133.9 ± 9.2	1304.7 ± 36.8

Pepina Linneusza	nd	nd	25.3 ± 2.6	<LOQ	197.1 ± 18.4	<LOQ	858.8 ± 36.5	68.1 ± 0.4	159.1 ± 9.1	1308.4 ± 36.5
Pepina Ribstona	nd	nd	19.2 ± 1.7	nd	194.7 ± 14.9	nd	834.3 ± 32.3	42.5 ± 0.2	204.6 ± 12.0	1295.3 ± 30.7
Piękna z Rept	nd	nd	nd	nd	141.3 ± 9.3	9.8 ± 0.2	580.2 ± 20.6	43.1 ± 0.3	27.3 ± 2.1	801.7 ± 25.2
Reneta Blenheimska	nd	nd	nd	nd	156.7 ± 11.2	17.6 ± 1.6	712.4 ± 29.1	39.8 ± 0.5	119.1 ± 7.7	1045.6 ± 31.6
Reneta Harberta	nd	nd	nd	nd	211.8 ± 14.5	22.3 ± 1.8	957.4 ± 33.7	71.2 ± 0.7	232.3 ± 17.6	1495.0 ± 42.0
Reneta Kanadyjska	nd	nd	nd	nd	118.2 ± 8.6	14.0 ± 1.1	516.6 ± 45.8	37.6 ± 0.4	68.9 ± 6.2	755.3 ± 27.1
Reneta Kulona	54.3 ± 5.1	nd	nd	28.1 ± 4.0	244.5 ± 21.4	13.9 ± 1.0	191.7 ± 22.2	nd	115.8 ±8.3	648.3 ± 25.3
Reneta Strauwalda	nd	nd	nd	nd	147.2 ± 10.1	21.6 ± 1.9	569.0 ± 34.3	42.4 ± 0.0	78.3 ± 11.0	858.5 ± 27.7
Reneta z Brownlee	156.3 ± 10.7	nd	nd	27.4	43.4 ± 5.4	36.6 ± 3.3	117.6 ± 9.5	nd	27.2 ± 2.5	408.5 ± 13.2
Schieblers Taubenapfel	nd	nd	11.9	nd	223.1 ± 19.9	20.5 ± 2.0	698.2 ± 32.0	<LOQ	109.4 ± 5.1	1063.1 ± 34.5
Szara Reneta	50.33 ± 5.7	nd	nd	nd	122.4 ± 11.8	13.9 ± 0.8	495.4 ± 27.9	14.2 ± 0.4	76.6 ± 12.2	772.8 ± 28.1
Złota Reneta	59.7 ±7.5	nd	nd	38.9	168.9 ± 16.7	8.4 ± 0.1	735.6 ± 31.1	52.8 ± 1.1	210.0 ± 10.0	1274.3 ± 31.9
Złotka Kwidzyńska	nd	nd	12.0	nd	202.7 ± 19.4	15.6 ± 1.2	692.9 ± 17.3	32.4 ± 0.7	125.3 ± 7.3	1080.9 ± 27.6

Table S7. Polyphenols identification. Abbreviations: RT_{exp}, retention time of compound found in extracts from DAP [in minutes]; RT_S, retention time of standard (if used) [in minutes]; RT, identification based on comparison of peak's retention times in sample and retention time of standard; UV-Vis, identification based on specific UV-Vis spectrum; NI - unidentified compound (lack of standard, partial identification based on specific wavelenght spectrum), *chlorogenic acid belongs to phenolic acids group but was analyzed with polyphenols, **phloretin (aglycone) was not present in extracts from DAP but was used as standard to quantification.

**Compound**	**RT_{exp}**	**RT_S**	**Identification**	**Quantity evaluated using standard**
Chlorogenic acid*	13.0	13.0	RT/UV-Vis	Chlorogenic acid
Epicatechin	15.1	15.1	RT/UV-Vis	Epicatechin
Quercetin-3-rutinoside (Rutin)	18.9	18.9	RT/UV-Vis	Quercetin
Quercetin-3-galactoside (Hyperoside)	20.5	20.5	RT/UV-Vis	Quercetin
Quercetin-3-glucoside (Isoquercitrin)	21.0	21.0	RT/UV-Vis	Quercetin
Quercetin-glycoside (NI)	23.0		UV-Vis	Quercetin
Quercetin-glycoside (NI)	24.1		UV-Vis	Quercetin
Quercetin-glycoside (NI)	25.4		UV-Vis	Quercetin
Phloretin-glycoside (NI)	25.8		UV-Vis	Phloretin
Quercetin-3-rhamnoside (Quercitrin)	26.2	26.1	RT/UV-Vis	Quercetin
Phloretin-glycoside (NI)	31.6		UV-Vis	Phloretin
Quercetin (aglycone)	36.1	36.0	RT/UV-Vis	Quercetin
Phloretin (aglycone)**		38.1		

Table S8. Comparison of polyphenols content in analyzed apple cultivars. Values in mg/100 g of DAP. NI - compound partially identified based on specific wavelength spectrum (lack of standard). Bolded cultivars with TPC higher than 200 mg GAE/100g of DAP. One-way ANOVA analyses shows statistically significant differences between cultivars (P < 0.05).

**Cultivar**	Chloroge-nic acid	Epicatec hin	Rutin	Hyperos ide	Izoquer-citrin	Quercetin-glycoside (NI)	Quercetin-glycoside (NI)	Quercetin-glycoside (NI)	Phloretin-glycoside (NI)	Quercitr in	Phloretin-glycoside (NI)	Quercetin (aglycone)	**Total content:**
Boskoop	180.8 ± 9.2	2.5 ± 0.0	108.5 ± 7.6	897.1 ± 45.7	91.1 ± 8.7	160.3 ± 8.7	34.6 ± 1.1	236.9 ± 20.2	59.4 ± 0.4	157.3 ± 7.6	214.6 ± 7.4	82.3 ± 6.6	2225.3 ± 79.2
Grafsztynek Inflancki	291.1 ± 10.6	2.3 ± 0.0	0.5 ± 0.0	50.0 ± 0.1	26.8 ± 0.2	17.2 ± 0.2	3.4 ± 0.1	26.5 ± 0.3	19.0 ± 0.2	29.0 ± 0.3	10.6 ± 0.2	2.4 ± 0.2	478.7 ± 30.5
**Grochówka**	168.7 ± 7.7	2.8 ± 0.0	110.1 ± 5.4	1512.3 ± 55.4	295.9 ± 14.1	412.5 ± 11.1	32.3 ± 1.2	841.2 ± 34.6	83.5 ± 3.4	612.0 ± 25.4	419.1 ± 8.7	230.8 ± 20.3	4721.0 ± 99.3
**Jakub Lebel**	144.0 ± 8.5	155.1 ± 5.3	7.8 ± 0.1	221.5 ± 11.2	126.2 ± 5.7	143.8 ± 7.5	7.5 ± 0.2	301.1 ± 14.4	47.6 ± 2.7	128.3 ± 5.7	167.0 ± 5.3	14.6 ± 0.8	1464.5 ± 46.6
**James Grieve**	258.3 ± 10.4	92.7 ± 4.2	179.1 ± 6.3	1715.9 ± 45.5	215.4 ± 10.4	336.4 ± 10.4	100.6 ± 1.9	615.1 ± 23.7	42.8 ± 3.6	252.4 ± 11.0	73.7 ± 2.2	16.1 ± 1.0	3898.6 ± 87.1
**Kantówka Gdańska**	364.8 ± 11.7	97.7 ± 5.6	101.4 ± 4.4	990.1 ± 32.1	299.8 ± 12.4	231.7 ± 7.9	19.1 ± 0.0	647.0 ± 32.5	269.0 ± 12.5	215.3 ± 9.6	1077.2 ± 39.3	14.2 ± 0.9	4327.3 ± 110.2
Kosztela	104.9 ± 6.0	11.5 ± 0.1	7.2 ± 0.2	518.0 ± 25.8	57.3 ± 0.6	129.5 ± 6.5	11.4 ± 0.1	243.2 ± 10.8	13.4 ± 0.0	227.6 ± 10.9	79.3 ± 2.1	19.9 ± 2.0	1423.3 ± 43.4
Kronselska	368.6 ± 11.9	nd	68.3 ± 0.9	614.9 ± 32.9	131.3 ± 1.2	154.1 ± 7.7	10.6 ± 0.1	356.9 ± 11.2	196.3 ± 9.7	177.1 ± 6.8	763.8 ± 27.9	134.1 ± 9.7	2976.0 ± 67.5
**Książę Albert**	100.2 ± 7.1	17.9 ± 0.3	0.7 ± 0.0	65.1 ± 4.4	15.3 ± 0.1	77.6 ± 5.3	5.7 ± 0.0	138.5 ± 9.0	133.5 ± 7.1	125.0 ± 7.1	165.1 ± 13.0	10.0 ± 0.2	854.6 ± 23.9
Książę Albrecht Pruski	36.5 ± 0.4	204.6 ± 4.7	13.6 ± 0.0	1165.8 ± 30.8	107.9 ± 2.1	301.0 ± 3.2	57.6 ± 1.5	679.2 ± 29.7	90.2 ± 3.1	202.9 ± 3.3	158.8 ± 7.7	21.2 ± 1.6	3039.3 ± 67.4
Niezrównane Peasgooda	49.9 ± 0.2	66.9 ± 3.2	21.0 ± 0.3	403.7 ± 14.1	90.3 ± 7.7	130.7 ± 5.2	20.5 ± 0.4	352.6 ± 17.7	236.3 ± 11.8	140.1 ± 7.1	321.2 ± 16.2	13.4 ± 0.8	1846.8 ± 56.0
Pepina Ribstona	204.3 ± 3.2	4.6 ± 0.1	6.5 ± 0.0	139.6 ± 4.5	38.1 ± 3.6	52.3 ± 0.6	8.7 ± 0.0	84.3 ± 5.3	101.5 ± 5.4	39.9 ± 0.4	133.9 ± 1.4	12.5 ± 0.7	826.1 ± 18.1
Piękna z Rept	296.0 ± 10.3	14.4 ± 0.1	256.3 ± 11.0	1152.4 ± 27.4	399.0 ± 14.8	300.2 ± 9.1	24.7 ± 0.1	508.8 ± 22.4	25.9 ± 1.1	363.8 ± 7.5	122.2 ± 2.5	93.3 ± 6.3	3557.0 ± 79.3
Reneta Kulona	481.2 ± 10.0	16.5 ± 0.1	53.6 ± 0.5	380.7 ± 9.0	122.9 ± 5.2	127.7 ± 5.0	11.5 ± 0.0	210.1 ± 10.1	56.5 ± 2.3	116.6 ± 4.2	245.2 ± 8.9	11.4 ± 0.1	1833.9 ± 50.2

Shieblers Taubenapfel	8.0 ± 0.0	4.1 ± 0.0	45.5 ± 0.6	1193.5 ± 19.7	214.7 ± 10.1	133.7 ± 4.9	nd	233.8 ± 6.5	150.5 ± 6.0	89.5 ± 1.9	164.5 ± 12.2	37.2 ± 3.1	2275.0 ± 47.1
Szara Reneta	97.5 ± 3.1	54.5 ± 0.6	nd	7.2 ± 0.0	2.4 ± 0.0	11.0 ± 0.3	0.3 ± 0.0	16.7 ± 0.2	88.1 ± 4.9	6.1 ± 0.0	726.7 ± 30.6	0.6 ± 0.0	1011.0 ± 26.8
Złota Reneta	219.3 ± 7.6	56.5 ± 1.4	50.9 ± 1.1	291.5 ± 15.3	93.7 ± 6.3	79.4 ± 4.8	9.4 ± 0.2	91.1 ± 0.7	184.2 ± 7.3	71.0 ± 3.5	612.9 ± 24.0	98.6 ± 7.6	1858.5 ± 21.9
